# Addressing Conceptual Randomness in IoT-Driven Business Ecosystem Research ^†^

**DOI:** 10.3390/s20205842

**Published:** 2020-10-15

**Authors:** Fabien Rezac

**Affiliations:** Interdisciplinary Centre for Digital Business Development (DBD), Department of Business Development and Technology (BTECH), School of Business and Social Sciences (Aarhus BSS), Aarhus University, 7400 Herning, Denmark; fabien@btech.au.dk

**Keywords:** ecosystem, business ecosystem, digital business ecosystem, IoT, internet of Things, IoT-driven digital business ecosystem, conceptual randomness

## Abstract

During the almost 27 years of its existence, the business ecosystem research has developed a substantial level of ambiguity and multifacetedness. Because to the technological advancements that promote interconnectedness and value co-creation, the field has consequently spun off into more domain-specific branches, such as the arena of digital business ecosystems that are driven by Internet of Things (IoT). Nonetheless, despite the efforts to mend the theoretical foundations and to close the gap between academia and empirical practice, the absolute majority of IoT-driven digital business ecosystem literature follows the trend of conceptual randomness while expanding the volume of publications exponentially. Therefore, in order to address this unfavourable increase in random adoption of distinct concepts that ultimately refer to the same subject matter, the author encourages other scholars involved in the research field of IoT-driven digital business ecosystems to make extended efforts and support the external validity of their research (as well as the relevance of the research stream as a whole) by bounding the IoT-driven digital business ecosystems on a rigorous basis through deploying the extant theory in a careful and appropriate manner. Via a thorough examination of the theoretical fundaments that underpin the concept of IoT-driven digital business ecosystem, and based on a concise thematic review of corresponding literature published until September 2020, this article articulates logic for viewing the conceptual hierarchy within the business ecosystem research and proposes six literature-based recommendations for developing further IoT-driven digital business ecosystem (DBE) research in a rigorous way.

## 1. Introduction

It has been a while since G.G. Leibnitz refined the system of binary numbers and came out with the well-known “Explication de l’arithmeétique binaire” [1]. Published approximately 270 years before the first electronic large-scale general-purpose digital computer entered the public domain [2], this article is considered by many to be the earliest antecedent of what is now commonly labelled as digitalization, i.e., “the innovation of business models and processes that exploit digital opportunities” [3]. Whether perceived as an overused buzzword or dogmatically praised as a condition to survive among the fittest, leveraging digitized data for the improvement of business processes has become a key success catalyst for many organizations and an invincible hurdle for others. Without a doubt, nowadays companies are in the position of having a chance to seize the vast opportunities of digital business development, such as seizing increased efficiency or adopting new business models [4]. Moreover, it has become increasingly inevitable to be engaged in any kind of business activity without coming across this future 21st century’s artifact. As humankind gradually reached the phase of the Fourth Industrial Revolution, businesses began facing a promising challenging transformation relevant to all parts of industries and society [5]. New technologies, such as artificial intelligence (AI), ledger technologies, or Internet of Things (IoT), all fuse the physical, digital, and biological worlds [6]. Ultimately, as all disciplines, industries, and economies are impacted, companies have been finding themselves under the pressure to rethink their value chains and change the way of how they conduct business [7]. Strategies that are based on alternative value propositions suddenly do not work anymore and a major paradigm shift towards modularity and complementarity disrupts the business landscape per se. From a perspective of a stakeholder that is directly involved in the deployment or consumption of such developments, it could be tricky to realize the magnitude of this upheaval in the making. The concept of business as such has been gradually yet radically moving away from the long-lasting state of affairs built on traditional structures and somehow shallowly conservative operational logic [8]. The prospect of sustainably prospering now lies in addressing much broader customer needs and it has become simply impossible for companies to cope alone and capitalize on their isolated supply chains [8]. Moreover, as the value creation and capture opportunities migrate towards the philosophy coopetition, a new cornerstone of gaining increased competitive advantage is being set in the form of finding new ways to collaborate without the necessity of hierarchical governance. Consequently, companies increasingly form interdependent networks of self-interested actors jointly creating value, i.e., business ecosystems (BE) [9].

As elaborated below, the use of the term “ecosystem” has been booming. In 2019, it appeared in annual reports approximately 13 times more frequently than a decade ago [10]. Nonetheless, the BE literature related to business research is on a large scale rather ambiguous and suffering from a limited degree of conceptual consensus. Because the exponential progress of technological development does not wait for academia to catch up, a digital layer has gradually been added on the top of virtually every aspect of business. This actuality made the researchers shift their focus toward exploring the newly emerged field of digital business ecosystems (DBEs) without determining the actual dimensions of its parent concept. Additionally, while the DBE research area is rooted in BE, none of them can be undoubtedly considered as rigid enough to keep up with the rapid pace of development in a coherent manner. For instance, by 2023, there will be roughly 3.6 devices connected to IP networks per capita. This sums up to 29.3 billion networked devices, i.e., 10.9 billion more than in 2018. Additionally, crucial to mention, approximately half of these devices will be IoT [11]. The capacity of smart and connected products is so extensive, it reshapes not only the mechanisms of competition within the industry, but also the underpinning definition of the industry itself, as Porter and Heppelman [12] stress. The interrelated products now allow companies to collaborate and propose value that cover a much larger, complex spectrum of customer demands. Each of us can be now considered a “walking data generator” [13] and the rise of data-driven technologies has affected the way how business is conducted [14] to the degree that some researchers even regard the current climate as “data capitalism” [15]. There is no wonder that the focus has been yet again shifting to investigation of a very specific area of DBEs—DBEs that are driven by IoT as one of the most significant game-changing technologies of the last decades. 

It is apparent that the interest in the IoT-driven DBEs has been increasing in a similarly exponential fashion as the interest in its parent concept of BEs. Moreover, based on the projections from the IoT domain, it is legitimate to expect the exponential growth trend of this research area to continue escalating even more. Nonetheless, a need to reflect on the fact that authors throughout the different territories of the BE research actually enforce different interpretations of what the subject matter is arises in parallel. Additionally, while some of the scholars demonstrate a strong dedication to mend the scattered shards of BE research and shape it into a dedicated unit of analysis that could serve as a solid base for further academic endeavours, such as focus on IoT-driven DBEs (e.g., [16]), it is apparent that a common understanding is still yet to be achieved. Based on the character of IoT-driven DBE research, i.e., DBE research that is driven by a specific technology, most of the researchers referring to this phenomenon are naturally coming from domains different than business and management. Therefore, not being necessarily aware of the fact that defining the concept of BE is an ongoing crusade that entails constant, iterative revisions, and also possibly unconscious of the cumulative impact of their actions, they tend to reach for low-hanging fruits in terms of easily accessible definitions that, however, may have already significantly advanced. Which is neither incorrect nor internally invalid. However, it is not beneficial for the field’s capacity to move forward as a whole either. This random deployment of different concepts that describe the same unit of analysis from different angles or levels of abstraction results in a counterproductive impact on the rigor of the whole field. Moreover, concepts are, in social sciences, generally known as one of the key building blocks of theories (e.g., [17]) and without a clear determination of what concept particular research projects work with, the whole stream is bound to perish or is even doomed to fade into a vague terminology that encompasses everything and nothing at the same time. 

Fortunately, the research on DBEs driven by IoT is still in a relatively early stage of development. Hence, it could be highly beneficial to seize the opportunity off assessing the variety of nuances among the extant notions toward the IoT-driven DBEs before the number of contributions skyrockets. For that reason, the following sections explore the very foundations of the BE research, tap into the fuzzy evolution of the concept that is caused by the emergence of a spin-off field driven by general digital technologies, and examine the conceptual status quo of a distinctive, but notably accelerating DBE research stream driven by the pervasive progression of IoT technologies. Consequentially, the article culminates in a concise discussion of the arising implications embodied in six literature-based recommendations for developing further IoT-driven DBE rigorously. Finally, the author reflects on a potential future research agenda, contemplating the challenge of achieving a balanced yet comprehensive level of interdisciplinarity that bridges the gap between different perspectives on the identical unit of analysis.

## 2. Fragmented Understanding of Business Ecosystems

The phenomenon of BE as a term was first recognized by James F. Moore back in 1993. In his classic Harvard Business Review article, called “Predators and Prey: A New Ecology of Competition” [18], the BE pioneer coined the term “business ecosystem” while pointing out the analogue between biological and business communities. Moore suggested that a company should not be perceived as a member of a single industry, but rather as an entity that is part of Bes, which cross a number of varied industries. He proposes that BEs progressively evolve from a phase of “random collection of elements to a more structured community”. In this community, the companies manage the complex interplay of cooperation and competition to generate value, which is superior to the value that could be created and captured by any of them individually. Quite possibly, this effect can be the most practically explained metaphorically in layman’s terms on a simple equation: 1 + 1 = 3. BE evolve in a continuum consisting of four distinct stages, i.e., birth, expansion, leadership, and self-renewal, according to Moore’s theory. Although these stages naturally overlap, each of them encompasses respective cooperative and competitive challenges that surface along their innovation journey. During the birth phase, the companies need to start collaborating across their supply chain vertical in order to establish the new value resting upon an emerging innovation. Nonetheless, at the same time, the novel ideas have to be protected and key stakeholders locked in. In the second stage, expansion, the cohort brings the offer to the market and focuses on scaling it while attempting to eliminate alternatives and making the service (or product) a widely accepted standard dominating the key segments. The following leadership stage is interlaced with leveraging visionary outlook to keep the BE members interested in the continuation of their cooperative engagement while maintaining the bargaining power over them. The fourth and final stage, self-renewal, is quite self-explanatory. To reinvent itself, the ecosystem has to be open to external innovation, while keeping the barriers of entry and switching costs high enough to ensure ample time to implement it. This idea closely corresponds with the logic underpinning the concept of “strategic drift”, as presented in Johnson et al. [19], i.e., change or die. Moore later refined the theory and presented his definition of BE in a book called The Death of Competition: Leadership and Strategy in the Age of Business Ecosystems [20].

The above paragraph describes the adverbial “big bang” of the BEs. Nonetheless, since placing its first stepping stone, the research has undergone a considerable evolution and increase in popularity. Therefore, there is a need to unveil a couple more determinant articles that will illustrate the essential basics exhaustively in order to get oriented in the snarl of BE literature. One of them is a paper called “Ecosystem as Structure: An Actionable Construct for Strategy” written by Ron Adner [21]. He proposes that BEs can be viewed either as an affiliation or as a structure. The ecosystem-as-affiliation approach is in line with Moore’s original understanding of this phenomenon and it is used by number of scholars. On the other hand, “ecosystem-as-affiliation” focuses mainly on the aggregated macro factors of BEs, providing an adequate lens for exploring links and interactions between the complementary actors and focal orchestrators. In Adner’s words, it “places emphasis on the breakdown of traditional industry boundaries, the rise of interdependence, and the potential for symbiotic relationships in productive ecosystems. It focuses on questions of access and openness, highlighting measures, such as number of partners, network density, and actors’ centrality, in larger networks. In the business context, analyses held at the level of the ‘healthcare ecosystem’, the ‘Microsoft ecosystem’, the ‘Silicon Valley ecosystem’, or the ‘entrepreneurial ecosystem’ fall easily into this category”. Nonetheless, he argues that this approach is not clearly distinguishable from other interdependence areas of research and does not encompass sufficient means necessary to facilitate understanding of the considerations linked to business model components. Adner’s alternative approach of “ecosystem-as-structure”, on the other hand, positions the interdependent value proposition in the very center and aims to recognize the multilateral set of interacting actors and their alignment structure that is required to capture the proposed value. The structure is defined by four key components, i.e., activities necessary for value capture, actors executing those activities, positions determining concrete location of actors in the dynamics of performed activities, and links that determine the transactions of more or less tangible resources that may or may not be channeled straight toward the BE orchestrator.

Adner’s approach bifurcates the discipline into an “either/or” situation. Approaching BEs from a slightly different angle, Jacobides et al. [22] acknowledge that Adner [21] offers “a view on how ecosystem research relates to established views and offered a useful guide to the differences in phenomenological emphasis between ecosystem research and other streams”. Nonetheless, they take a step further and synthesize “existing research in order to elucidate the key mechanisms behind the emergence and dynamics of ecosystems, and why we have seen such a rise in interest”. In that spirit, they take into account that different researchers adopt different aspects of ecosystem as the central unit of analysis, dividing the research contributions into three streams: business, innovation, and platform. These revolve, respectively, around firm and its environment, particular innovation or new value proposition, and the constellation of actors that support it or considerations of how actors organize around a platform. However, in reality, all of these aspects overlap. Therefore, in order to progress sharply, there is a need to build future research coherently on a consensual understanding of this phenomenon. Jacobides et al. hence suggest that the definition should encompass three key BE attributes, i.e., multilateral, nongeneric complementarities, set of roles that link the individual actors and no unilateral hierarchical control, and frame BE as “a set of actors with varying degrees of multilateral, nongeneric complementarities that are not fully hierarchically controlled”. These actors create higher value together than any of them would be able to create individually. According to the authors, the emergence of ecosystems is possible due to the modular architecture that allows for the organizations to be coordinated without “full hierarchical fiat” and keep their autonomy. The balance between interdependence and independence is a common denominator of the majority of ecosystems in many different industries.

While the contributions by Jacobides and Adner are by many considered to be the most momentous in recent history, Bogers et al. [9] suggest that, instead of narrowing the concept down, there is a prevailing need for a definition that is much broader and integrates all of the BE streams into a truly comprehensive framework. Thus, in their Academy of Management Proceedings paper, the authors oppose Adner [21] by arguing that both memberships and structure of BEs should be considered. Moreover, they tackle the narrowed focus on strong network complementarities proposed in Jacobides et al. [22] by developing an overarching perspective that summarise the key feature of the BEs and defines a BE as “an interdependent network of self-interested actors jointly creating value”. Based on an extensive analysis of the BE research since its very origination, this definition addresses the lack of validity and poor unclear conceptual boundary conditions by identifying three operational constructs: goals of ecosystem members, the network of relations between these members, and the interdependence of their respective goals. This is linked to the key purpose of BEs as such—Joint value creation. The construct of ecosystem members’ goals consists of firms sponsoring the ecosystem, other member firms, non-profit organizations, and individuals. These members have different motivations and, although they usually tend to work toward the cumulative success of the ecosystem as a whole, barely any entity in business is purely altruistic and it is natural that their self-interests are of higher priority. Thus, it is crucial to understand their underlying motivations to join the ecosystem as well as the why behind them. Interdependence between members is concerned with the dynamics of the relationships between the involved members, who are all invested in the success of the BE. This interdependence can be cooperative, competitive, or coopetitive, i.e., both cooperative and competitive at the same time, as described in by R. Kapoor and J.M. Lee [23]. The final construct, called network structure and governance, then examines the considerations implied by its appellation, i.e., “the collective arrangement between the interconnected members”. The research agenda drawn up in this document then clearly, in detail, identifies what is needed to unfold in order to really move forward—respective constructs, linkages between them and, finally, the BE as the unit of analysis by itself. This paper provides a very thorough starting point for understanding the basics of the BE field and it can definitely be considered to be among some of the most overarching overviews written so far. 

It is crucial to mention that in the abovementioned article “Towards a theory of ecosystems”, Jacobides et al. also identified three groups of ecosystem papers: business ecosystem stream, innovation ecosystem stream and platform ecosystem stream [22]. Additionally, although the terms used in each of the respective streams are closely interrelated, they are all built on different foundations and should not be used in an interchangeable manner. The first stream of literature (in which also belongs to this very document) puts a firm and its environment in the centre of focus and views the BE as a “community of organizations, institutions, and individuals that impact the enterprise and the enterprise’s customers and supplies” [24]. Hence, the stream that examines BE views an ecosystem as an “economic community of interacting actors that all affect each other through their activities, considering all the relevant actors beyond the boundaries of a single industry” [22]. The second stream of literature revolves around a particular innovation or novel proposition of value and the arrangement of actors that facilitate it. While emphasizing the interactions between interdependent actors to create and commercialize value, this literature views the ecosystem as “the collaborative arrangements through which firms combine their individual offerings into a coherent, customer-facing solution” [25]. The third stream of publications studies platforms as a specific type of technology and investigate how the interdependent sponsors and their complementors organize around them. The scholars that engage in the discourse of platform ecosystems view the phenomenon as comprising of “the platform’s sponsor plus all providers of complements that make the platform more valuable to consumers” [22]. Followingly, the platform ecosystems are being understood as multisided markets or semi-regulated marketplaces. 

The advancement of BE research itself evinces some traits of linear continuity, as demonstrated in Table 1, below. Nonetheless, on a large scale, the literature is overall rather ambiguous and suffers from a limited amount of consensus on what a BE actually is. As a unit of analysis that is central to a stream studying real-life phenomena, BEs certainly do not exist in vacuum. In fact, the contrary is true.

## 3. State of Consensus in Digital Business Ecosystem Research

In order to understand the background of DBEs, it is first necessary to put the BEs in the context of the real world, as indicated in the previous section. Looking at BE from a macro perspective, the recent economic developments significantly impacted the emergence of BE. In his 2019 Harvard Business Review article, called “In the Ecosystem Economy, What’s Your Strategy?” [7], Jacobides draws up the three main structural changes in economy that are directly linked to the increased importance of BEs. Firstly, regulations are changing, and digitalization allows for increased modularity in business structures, leading to the development of brand-new component recombinations. Therefore, otherwise independent suppliers bundle unrelated products and services together, which makes the line between them less concrete. Secondly, regulations that used to protect the companies’ advantage of being exclusive in addressing certain customer needs are fading away and the integrated offerings now cover cross-domain areas. Lastly, technological progress is shifting the way companies can provide value to customers. Hence, the complementing products and services are matched and delivered in a previously unimaginable manner. For those reasons, the value created by individual firms can only just keep up with the value generated in BEs. In fact, the gap between them is getting so wide that, in certain sectors, single firms (or even industries) are not regarded as relevant subjects of analysis anymore. Therefore, the strategic considerations that involve BEs must focus on different aspects than those dealing with isolated firms, e.g., needs of complementors [7]. Overall, it is important to acknowledge that there is no recipe for a successful BE management. However, what is certain is that, without a working business model, no strategy can work [26]. Therefore, all of the actors must take care of their extant revenue sources when getting involved in external BEs.

In order for a BE to prosper, it is absolutely crucial to strike the balance of satisfied value capture interests of actors that must complement the value proposition that is based on various and seamlessly integrated customer services. There are, of course, instances of BEs centred around monopolistic entities (e.g., Facebook, Google), which developed the value proposition so attractive that complementors basically have no choice than to follow their lead and satisfy the orchestrator-influenced needs of their customers. In these extreme cases, the orchestrators can do whatever they please; however, in any healthy ecosystem, value capture and value proposition should be maintained in balance [27]. Moreover, ecosystems can bridge the public good and private benefit [8]; hence, it is of interest for the whole society to change the single-firm-centric analytical foundations of the national-level competition law and regulations. It is important to mention that not every company is meant to be the orchestrator of a BE and poorly evaluated ambition of becoming the central architect instead of an ordinary actor leads to a rather damaging outcome [8]. To become the leader, the firm must be in possession of a superior product or service that is hard to replicate, a strong brand, and a vast network of users. Another key role is played by the alignment of cultures and long-term goals, allowing for companies to co-orchestrate business ecosystems or at least buy in their way through investments. Otherwise, it might be more beneficial to create higher value just by taking part rather than taking over. Even a larger company that is capable of competing with its rivals can choose to enter their ecosystem as a complementor instead of being in the position of counter leader, endangered by unnecessary disruptions imposed by outside parties. This way, it can gain insights, understand the customer base, improve its skills in orchestration, or even open the doors to new markets, while keeping the core business away from major threats.

From the orchestrators’ point of view, it is also necessary to focus on finding a balance between optimal value-creation and number of ecosystems managed. Additionally, despite the fact that business ecosystem governance research is still in a relatively early stage, it is certain that orchestrators have to make decisions regarding the degree of the ecosystem’s openness and the attachment of the complementors. Furthermore, as Teece [28] proposes, the value being captured is dependent on the dynamic capabilities of the firms, scarcity of their respective resources, nature of their complementarities, and their adopted business models. By the means of expanding his original Profiting from Innovation (PFI) network, he stresses the importance digital platform-based ecosystems and argues that the abovementioned dynamic capabilities allow the “platform leaders”, i.e., orchestrators, to capture from innovations through designing suitable business models and building ecosystems. Helfat and Raubitschek [29] build on his theory and argue that, for the platform leaders, three types of dynamic capabilities are necessary: integrative capabilities for ecosystem orchestration, innovation capabilities, and capabilities to scan and sense the environment.

For the sake of reinforcing the common ground before entering the world of digital, it is also necessary to present the key myths of business ecosystems dispelled by Fuller et al. [10]. As the ecosystem gradually became a buzzword, it might seem that engaging in a BE also became a necessary condition for being successful. However, not every issue can be addressed by BEs. Hence, before establishing one, it is of substantiality to determine its purpose. Subsequently, it should be made generally clear that BEs are not another label for a supply chain. Viewing BEs as a supply chain is myopic, shrinking the research potential of the field. BEs reach beyond supply chain that can certainly be part of it but does not have to play a role at all. Furthermore, although all BEs have to be to a certain extent open, it they do not have to welcome everyone. Additionally, absolutely necessary to highlight is the fact that BE does not equal digital platform. Technology can play a key role in BEs existence; nonetheless, it is a means of orchestration or a form in which gradually evolve. Moreover, becoming a part of a BE is not only a matter of external relationships. Especially as the world experiences the Fourth Industrial Revolution [6], the data-driven BEs require internal processes to become more flexible and increasingly responsive. Based on that, it has to be acknowledged that BEs are complex and dynamic—Anything but static. Additionally, to seize the emerging opportunities, BEs must be viewed in that way. One of the myths is also that any actor can be the orchestrator. However, as mentioned in previous paragraph, to co-create and co-capture value is demanding of particular assets that not every firm has at disposal. Additionally, building on its key characteristics embodied in the definition, BEs can be only partially controlled. The interactive nature of co-evolution cannot be planned and executed. This means that determining strategy to follow is also not a prerequisite to yield success. On the contrary, being capable to shape strategy in accordance with occurring situations is much more determinant.

It is already apparent that BE is a tricky phenomenon to grasp—Especially for scholars from domains other than social and business sciences. Thus, adding the digital layer on top is an ideal trigger for a great deal of confusion, which could easily spill over into the offshoot stream of DBEs and possibly even further. Examining the origins of DBEs, the term itself was first used also by Moore, in 2003 [30]. Nonetheless, surprisingly enough, it was used in an unrelated context. Hence, the credit for igniting the debate in the relevant area of interest is attributed to Nachira et al. [31], who, in his document for European Commission, emphasized the “the coevolution between the business ecosystem and its partial digital representation” and described DBEs as an environment of collaboration consisting of different actors that co-create value using information and communication technologies. It is obvious that DBEs can be viewed not only as the aforementioned concepts or an EU project, but also as a technology [32]. Over the years, the DBE concept has been deployed in an extensive number of academic and industrial disciplines, including management, information systems, computer science, and tourism, as evidenced from a recent extensive and highly cited literature review by Senyo et al. [33]. The current literature splits up into four thematic streams, which consist of a number of sub-themes identified by axial coding. The first stream is concerned with the business issues and focusses on the commercial and value co-creation aspects of DBEs, including alliances, network analysis, value co-creation, governance, legal issues, knowledge development, dissemination, strategy, processes, management, trust, risk, and security. The second stream explores the technological side of DBEs, including platform design, technologies, architecture, systems integration, interoperability or process, and service design. The third stream deals with DBE conceptualizations and involves the sub-themes of DBE projects, development, management, genesis, and properties. The fourth and last stream of DBE literature is dedicated to DBE artefacts, such as methodologies, models, modelling languages and frameworks. All four streams entail a whole spectrum of potential research directions that should be pursued, cumulatively forming a thorough research agenda stressing the key pain points that deserve attention so the field can move forward in balance. 

To sum up the findings of Senyo et al. [33] that are presented in the previous paragraph, the DBE research itself can be in a sensible way divided in approximately 30 different aggregated clusters that examine different aspects of the phenomenon from different angles. However, not surprisingly, the absolute majority of the studies actually focus on a different unit of analysis. The polymorphous nature of the subject allows for scholars to choose their position on an interpretative basis, which makes the field even more intangible and diverging from reality. This statement can also be supported by bare facts. Almost shockingly, more than half of the publications are of conceptual substance. This means that a majority of findings have never been tested for their actual applicability in real life. What is equally shocking is the fact that almost three quarters of the articles have no theoretical grounding. Therefore, despite the obvious far-reaching importance of DBEs has been moving away from practice, while those who are still interested mainly engage in conceptual discussions without almost close to none theoretical grounding. Not addressing this gap may lead to further trivialization of the field, essentially making all of the studies internally valid, but increasingly useless in the broader context. Developing the field in this manner or using it as a foundation may be not only counterproductive but would also elicit an unwanted occurrence resembling logic behind a legal metaphor, called “fruit of the poisonous tree”. This phrase, coined by Justice Frankfurter in 1939 in the case Nardone v. United States, stands upon a doctrine that was established in 1920, by the decision in the case Silverthorne Lumber Co. v. United States [34]. In essence, this parallel suggests that if the primary source is tainted, everything that is based on that source is tainted as well. Unfortunately, considering the interdisciplinarity of DBEs specifically, these poisoned fruits are hanging fairly low.

For illustration, when searching the articles using “digital business ecosystem” as a query, a paper formulated by G. Del Chiappa and R. Baggio, called “Knowledge Transfer in Smart Tourism Destinations: Analyzing the Effects of a Network Structure” published in the Journal of Destination Marketing & Management, pops up as the most cited (99 citations, according to Scopus) [35]. This journal “aims to be the leading international journal for the study of tourist destinations by providing a critical understanding of all aspects of their marketing and management, as they are situated in their particular policy, planning, economic, geographical and historical contexts” in order to provide a more thorough context. Moreover, it explicitly “seeks to develop a robust theoretical understanding of destination marketing and management by harnessing knowledge drawn from the full breadth of disciplinary approaches to the study of destinations”. Based on that, it is quite clear that, although the main interest of this article is to advance the field of destination management, it also possesses conspicuous features of interdisciplinarity. Despite having “digital business ecosystem” stated as the first keyword of this article, it barely touches upon the definition of this concept. The authors present Nachira et al. [31], an EU document, as the key reference and mention it only superficially. Moreover, this reference belongs to the sub-stream of research studying DBE properties and origination that suffers from the absence of a “thorough discussion of related concepts such as BE, collaborative network and innovation ecosystem”, resulting in “confusion in the literature as to the differences and similarities between DBE and related concepts” [33]. In practice, this could mean that, when searching for a reference regarding DBEs, scholars from the destination management domain could easily rely on this otherwise highly reliable and valid article and use it a main source of DBE authority, thus increasing the incidence of the term in their domain while debasing the cumulative value of the concept as such. When considering the far-reaching scope and cross-domain nature of DBEs, harvesting the low-hanging poisoned fruits can increase the popularity of conceptualizations that do not embody the main aspects of DBEs making the studies cut corners and ignore DBE’s immense potential to grow into a fully-fledged, standalone field of research. Naturally, this goes against the effort of the researchers fighting for embracing the diversity and building a cumulative rigor. For that reason, it is necessary to explore the extant situation of approaches toward BEs and DBEs within the realm of IoT-oriented studies.

## 4. Methodology

### 4.1. Research Design

The author has decided to enhance this position paper–based article by embedding a concise literature review that is explicitly focused on definitions of IoT-driven DBEs used in the peer-reviewed literature. The purpose of the review is to enfold the current conceptual status within the emerging, but unexplored, area of IoT-driven digital business ecosystem research. It is important to mention that, when considering its shallow depth and restricted scope, this section should be understood rather as a supporting argument-building component of this position paper–based article, carried out with the aim of gaining initial overview of the discussed research and related scholarly dialogues. This step then allows for identifying or disproving the potential legacy stigma of conceptual randomness that is rooted in the limited consensus within BE and DBE streams.

Despite the fact that only a “little methodological advice exists beyond generic and/or editorial guidelines”, no effort to “consolidate, organize, and synthesize the existing knowledge” should be haphazard, regardless its magnitude [36]. For that reason, the author has decided to conduct the abovementioned systematic literature review feature that was included in this article in line with the definition and process proposed by Kitchenham [37], i.e., “identifying, evaluating and interpreting all available research relevant to a particular research question, or topic area, or phenomenon of interest”. This extensively cited guideline has been originally developed for the researchers from the area of software engineering and it stands on the foundations that were composed of three major systematic review guidelines, i.e., The Cochrane Reviewer’s Handbook [38], Guidelines by the Australian National Health and Medical Research Council [39,40] and CRD guidelines for individuals conducting or commissioning literature reviews [41]. The process of the review is followingly divided into three phases that consist of respective stages, as indicated in Table 2.

The need for conducting this brief review has been based on the fact that no review of literature has yet been conducted in this area. Therefore, this step has been considered key and necessary in order to be able to draw a conclusion about the discussed phenomenon and move the debate further. The protocol has been developed on a pragmatic basis to serve the rationale of this article and determine the state of conceptual maturity within the emerging IoT-driven DBE field. First, procedure-wise, the author carried out an initial literature search to map the topic-related keywords, themes, and possible expert terms. Followingly, in order to construct queries involving Boolean operators, suitable keywords were selected and the actual searches processed while using the “largest abstract and citation database of peer-reviewed literature”—Scopus by Elsevier [42]. As far as the inclusion and exclusion criteria are concerned, the types of reviewed literature was limited to the main sources of new knowledge, i.e., articles, conference papers, books, and book chapters, from the year when the respective term emerged (i.e., 1993 for BE, 1999 for IoT and 1999 for BE intersected with IoT) until September 2020. Furthermore, the author has included articles written in English only. This approach has been deemed thorough enough to fill in the missing gaps for building up a sufficient mandate to discuss the arisen findings and propose their implications. The results were synthesised on the basis of thematic analysis, which is a is a method for “identifying, analyzing, organizing, describing, and reporting themes found within a data set” that, despite no consensus about how can the researchers apply the method rigorously, can generate “trustworthy and insightful results”, in order to support this position paper in a suitable way [43,44]. The details of specific searchers are available in Section 4.2, while the synthesized thematic findings are presented in Section 5.3. 

### 4.2. Search Overview

The first search round was conducted in order to explore the trend of the BE and DBE entries since the moment Moore defined it in 1993 [18]. As some authors refer to BEs and DBEs simply as “ecosystems”, this keyword was also included. Besides fluctuations caused most probably by the ending of a specific EU project called “Digital business ecosystem” [45], the ecosystem topic in the area of business and management has been receiving substantially increasing attention, i.e., looking on the status in the five-year interval, 14 documents were published in 1998, 32 in 2003, 153 in 2008, and 386 in 2013. Moreover, 947 documents were published in 2018, 1146 in 2019, and 883 already in 2020, as of 16 September 2020. A similar trend is also observed in the theme of IoT. Since the inception of IoT definition in 1999 [46], the term was in the business and management literature mentioned in 3273 documents with 1 relevant peer-reviewed document published in 2004, eight in 2009, 102 in 2014, 1301 in 2019, and 607 in 2020, as of 16 September 2020. 

However, the most important round of the conducted literature review is examining the correlation of the two abovementioned arenas. Since both of these terms had a chance to co-exist (i.e., in 1999), their concurrent use in business and management literature has been recorded in 155 relevant peer-reviewed instances, with the first relevant document in 2012: “Towards a Hyper-Connected World” by Marina Settembre [47]. It is interesting to note that 66.4 percent of the literature relevant to the business studies has been published in journals that are dedicated to subjects other than business and management, e.g., engineering, decision sciences, economics, econometrics, finance or mathematics. Based on that, it can be concluded that the stream of IoT-driven DBEs has been predominantly shaped by scholars from areas other than business and management. It can be observed that 44.5% of all of the publications are articles, 44.5%, 9% are book chapters, while approximately 2% are books. The overall increasing trend of relevant peer-reviewed published documents implies that the BEs and DBEs driven by IoT have been receiving more attention of business and management researchers with every passing year. 

**Figure 1:**
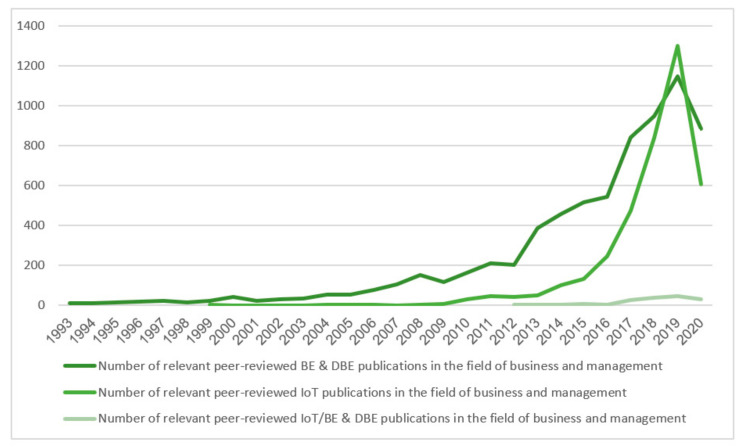
Relevant peer-reviewed literature search results.

It is important to mention is that the time span has been purposefully selected from the year when the respective concepts were established. Exploring conceptual consensus within a particular literature would not make sense without the key concepts still waiting to be introduced. Overall, the overview of the literature results, including used queries (without amendments to filter document type and year span), is presented in Table 3, while Figure 1 illustrates the development of the relevant peer-reviewed literature.

## 5. Towards Conceptual Consensus in IoT-Driven Digital Business Ecosystems 

### 5.1. Conceptual Hierarchy within the Business Ecosystem Research

**Figure 2:**
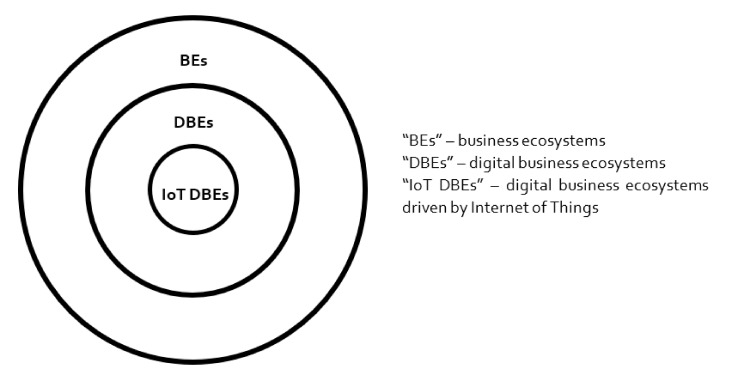
Proposed logic of conceptual hierarchy within the business ecosystem (BE) research.

As described at the beginning of this article, the most comprehensive, pragmatic and state-of-the-art understanding of BEs can be bounded into nine words: “interdependent networks of self-interested actors jointly creating value” [9]. This definition is certainly not dogmatic; nonetheless, it overarchingly comprises all of the important elements that BEs embody so far. Based on this, the conceptualization of DBEs should be theoretically built on this relatively common understanding. In that sense, DBEs as a stream of research should be ideally viewed as studying interdependent networks of self-interested actors jointly creating value, enabled by digital technologies. DBE is a type of BE that is distinguished by certain characteristics—as much as a car is a type of vehicle. Furthermore, IoT-driven DBE is a type of DBE that is distinguished by a particular type of technology that enables its existence. In that sense, a hydrogen vehicle is a type of vehicle with particular, unmistakable features distinguishing it from other cars. Nonetheless, as much as a hydrogen vehicle is still a specific type of car, which still is a specific type of vehicle, IoT-driven DBE is still a specific type of DBE, which still is a specific type of BE (see Figure 2). In a nutshell, all of the studies involving IoT-driven DBEs should be to a relevant level constructed upon theoretical foundations of BEs. This pattern of deductive reasoning then suggests that scholars who come across DBEs that are driven by IoT should treat them as an interdependent network of self-interested actors jointly creating value, being enabled by a specific type of digital technology, i.e., IoT.

Without embracing this structured approach, there is very little chance to make the BE, DBE, and IoT-driven DBE research streams solid enough. Imagine studying the market of hydrogen cars without considering the concept of a hydrogen car as a whole and predominantly focusing on certain aspects that only cover a fraction of features that together form the vehicle. The debate could be internally valid, but would not be beneficial to anyone.

### 5.2. Evaluation of Current Conceptual Consensus in the IoT-Driven Business Ecosystems Field

Based on the fragmented understanding of BEs and DBEs, it can be presumed that their core drawbacks that are presented in previous sections are also transferred on the studies that build on these concepts and put them in the context of IoT. For that reason, the author explored the set of 155 documents derived from the third search and identify how the respective authors approached bounding the definition and on what grounds their understanding stand. Although all 155 documents were subjected to the conceptual scanning, the author decided to only present the particularities of the first 20 in order not to congest the article with unnecessary details. This overview should be sufficient enough to illustrate the tangibility and traceability of the evidence, while allowing for the designation of the repercussions on firm ground. The list of the first 20 elaborated results is presented in the following paragraph, ordered from the most cited, hence, most influential and impactful article in a descending order. The complete list of articles without detailed content description but including DOI identifications, can be found in Appendix A.

1. Gretzel et al. [48] rely on a tourism- specific definition that sees the business ecosystem as one of the components of smart tourism. They reference Buhalis and Amaranggana [49], who build their conceptualization on Egger’s technological angle that investigates the ecosystem of “near field communication” [50].
2. Santoro et al. [51] focus on building a knowledge management system for open innovation and knowledge management capacity through IoT and refer to Soto-Acosta and Cegarra-Navarro [52], who do not employ any ecosystem theory at all.
3. Scuotto et al. [53] discuss the topic of smart cities and build on the innovation ecosystems in Schaffers et al. [54], who explore “smart cities as environments of open and user-driven innovation for experimenting and validating Future Internet-enabled services”.
4. Del Chiappa and Baggio [35] study the context of smart tourism and build their research on Nachira’s definition of DBE [32].
5. Rong et al. [55] focus on understanding business ecosystem using a 6C framework in Internet-of-Things-based sectors and base their research on the original definition developed by Moore [18].
6. Buhalis et al. [56] explore technological advancements from a value co-creation point of view in order to provide insights into service innovations that impact ecosystems. Focusing on tourism and hospitality, the authors stand on the medley of domain specific papers and describe “technology-driven platforms” as constituents of tourism ecosystems and “technological advancements” as an enabler of co-creation.
7. Baladina et al. [57] write about IoT use cases in healthcare and tourism and only mention ecosystems of IT services as a subject of change imposed by IoT as such, however without any theoretical grounding.
8. Papert and Pflaum [58] attempt to develop a theoretical ecosystem model for the implementation of IoT services in supply chain management. They present a review of literature, stress that the ecosystem research is multifaceted and continue in the research stream of Iansiti and Lakhani [14], Porter and Heppelmann [12], and Rong et al. [55]. As mentioned, Rong et al. [55] relies on Moore [18] and Porter and Heppelman [12], as well as Iansiti and Lakhani [14], and do not work with any explicitly framed ecosystem theory whatsoever.
9. Shin and Jin Park [59] propose that “an ecosystem, by definition, is a complex web of interdependent agents and relationships among people, technology and industry”. Nonetheless, their statement has no theoretical foundations at all.
10. Hein et al. [60] conduct an analysis of the value co-creation practices in a B2B platform ecosystem. With a focus on service platforms, they state that the “concept of ecosystems has changed the view from traditional inter-firm competition to a joint approach of coopetition”, while referencing Moore [18] (i.e., an affiliation-centred concept), Adner [25] (i.e., one of the works upon which a structure-centred concept is built, as explicitly mentioned in [21]), and Pereira et al. [61], who talk about coopetition and co-innovation in the context of service providers and manufacturers.
11. According to Uchihira et al. [62], “although many scholars have mentioned the structure of business ecosystem, concrete business-ecosystem design methods are not yet established, especially for IoT service”. Thus, using examples of smart factory and smart home, they propose a specific method of doing that. Their point of departure is Rong et al. [55], therefore rooted in Moore [18].
12. Rajput and Singh [63] come also from the IoT perspective, i.e., “IoT ecosystem consists of physical objects which are connected and accessible over the internet”.
13. An article by Madaan et al. [64] takes an entirely technical position and works with the term “(heterogeneous) IoT ecosystem” in connection to privacy threat of information linkage and different technical and legal ways of addressing it. The article contains no significant link to the business and management literature at all.
14. Although Harwood and Garry [65] mention Moore, it is only in relation to trust and changes in its nature depending on the agency of human actors and machine objects. While mentioning Iansiti and Lakhani [14] or Porter and Heppelman [12], they view ecosystems from a pure IoT-centric angle, i.e., “example of a techno-service system that renders synchronized actions for end-user consumption”.
15. Kummitha and Crutzen [66] discuss smart cities and citizen-driven innovation and mention ecosystem only as a general term linked to entrepreneurship and innovation.
16. As can be observed from the first sentence of their article, Suri et al. [67] are also purely technical: “The proliferation of connected devices, wherein Sensors, Actuators and Tags (such as Radio-Frequency Identification (RFID)) are able to seamlessly communicate to their environment and to each other for sharing information or to perform some actions has created the Internet of Things (IoT) ecosystem”.
17. Considering its specific focus on service domain, the research article by Chandler et al. [68] appears to have solid theoretical grounding. The authors have acknowledged some of the differences in the innovation- related ecosystem literature, use multiple sources and build the narrative in a well-anchored manner, sufficiently serving the purpose of their document.
18. Tesch et al. [69] mention IoT ecosystem rather as a context without considering it as a concept. They regard its complexity as a business-related challenge of IoT in general.
19. A paper by Leminen et al. [70] contains a whole section dedicated to the business models and ecosystems. The audience can clearly see from where are the authors coming and what concepts is their research built on. The story is unfolded gradually and in a rigorous manner.
20. Although the already mentioned article by Settembre [47] views ecosystems in a technical light, it identifies the changes in relationships and environment, pointing out the emergence of new opportunities as well as challenges. Nonetheless, the concept of ecosystem is introduced without sourcing any theoretical foundations.

### 5.3. Literature-Based Recommendations for Developing Rigorous IoT-Driven DBE Research

Borrowing concepts from other fields as such is far from unproblematic and it can even foster the progress of knowledge-creation. As the most obvious example can serve the BE itself – ecosystem as a biological concept provides an almost ideal analogy to a social-scientific phenomenon, sufficiently highlighting all of its important features, processes, and patterns that would be otherwise much more challenging to explain. Nonetheless, using concepts that were established in other scientific branches without proper consideration also entails a risk of increasing the unerudite application of those very terms, leading to creation and acceptance of hybrid concepts that lose on value with every unwittingly inapposite citation. Therefore, the authors synthesise the observations stemming from the identified patterns of drawbacks identified across the publications and proposes following set of six literature-based recommendations for developing IoT-driven DBE research in a resilient way in order to ensure a rigorous evolution of the IoT-driven BEs.

#### 5.3.1. Recognize Business Ecosystem as a Legitimate Concept

It is apparent that the DBE is gaining conceptual recognition, especially in the fields that are, by their nature, inclining to explore it as a dedicated unit of analysis, e.g., business and management. Nonetheless, when adapted by scholars from other areas, DBEs are approached rather as a general term with rather vague or even completely absent ontological grounding. Therefore, for the sake of nurturing the future evolution of the concept, the authors borrowing the term should recognize it as a legitimate concept and respect its theoretical fundaments.

#### 5.3.2. Acknowledge Its Multifacetedness

The boundaries of DBE are still in the process of being shaped and refined, which implies that it should not be approached dogmatically or as an absolutely self-contained entity. Leaning adamantly on just one school of thought might introduce unwanted bias to the entire research project, consequently limiting its potential impact. Therefore, the researchers should be familiar with the fact that different scientists interpret and approach BE from different perspectives; therefore, what could be valid in one domain might not be as on-point in the other. Ultimately, a single generally accepted overarching definition remains yet to come and, when dealing with DBEs, researchers should pay increased attention and spare no effort to be constructively critical.

#### 5.3.3. Revisit the Origins

Understanding the whole story behind DBEs before immersing into a specific instance of DBEs that is enabled by a particular technology, such as IoT, is quintessential for keeping the rigor alive. Using the DBE term should ideally be accompanied with acquiring insights regarding the original ideas that underpin its current state of affairs. Being engaged in the IoT-driven DBE research entails being focused on a very distinctive part of a far-reaching BE arena. Getting familiar with the foundation concept while subsequently exploring the evolutionary continuum in an offshoot stream that is enabled by digital technologies allows for being precautious against accidental misinterpretations of the key assumptions.

#### 5.3.4. Explore the Latest Developments

When tapping into DBEs, researchers should always attempt to be familiar with the latest developments and juxtapose the findings with the extant literature—not only in the area of IoT—driven DBEs and DBEs, but also BEs in general. The field of IoT-driven DBEs is in itself principally very much grounded in interdisciplinarity, as can be concluded from the conducted searches. Additionally, since most of the interest springs predominantly from the technology-focused area, it becomes steadily more exacting to keep up with the latest developments emerging in the social science. Consequently, the literature being in increasing volumes published on the topic of IoT-driven DBEs is, in a large part, built on definitions that have been already revised, updated, or synthetized into more suitable and improved formulations. Thus, this practice leads to a scattered development of the area and makes it rather intricated for it to progress as a whole and in the same direction.

#### 5.3.5. Determine the Definition to Be Applied in a Rigorous Manner

The researchers should be aware that there is not one single definition for a BE, DBA, or IoT-driven DBE. Additionally, the common understanding remains fragmented despite a number of concrete aspirations to establish a widely accepted and overarching umbrella concept. Before using the term, the researchers hence could find considerable benefits in getting oriented in the range of previously employed interpretations and approaches. The advantage of “knowing the ropes” can especially come in handy when determining the research angle to fit the particular topic of interest and designing appropriate methodology. Therefore, before making a contribution where any type of DBEs is meant to play even just a minor role, it is recommended to always skim the related state-of-the-art literature reviews and decide what up-to-date definition is the most suitable.

#### 5.3.6. Be Explicit about the Extent of the Intended Contribution

The researchers should invest time in contemplating whether and how much they actually intend to contribute to the narrative originating back in 1993. The use of the discussed terms with weak or without any theoretical substantiation leads to marginalization of the IoT-driven DBEs, resulting in the dissemination of vague misinterpretations making the field to expand in size but decrease in value. Therefore, this implies that the terms should be used purposefully. When relating to BE research in any shape or form, the researcher should carry out theoretical “due diligence” and explicitly state what school of thought is applied. On the other hand, the expert audience should be vigilant with regard to the occurrence of the term as a loose metaphor that is unrelated to the BE, DBE, or IoT-driven DBE research streams. Using those as the pivotal references for further research makes it unnecessarily compromised, essentially externally invalid, and diminishing the stream per se.

## 6. Discussion

To begin with, as referenced in the first paragraph, this article is an expanded version of a position paper presented on a Global Internet of Things Summit (GIoTS) 2020 [1]. In essence, the purpose of a position paper is to present an arguable opinion on an issue and explain the rationale behind that stance. Importantly, the position needs to be supported by evidence, critically evaluated, and followed by suggestions for the course of actions. For that reason, this article may seem of a rather unusual format. The author’s main motivation has been to identify and examine the key issues that emerge by colliding the two distinct yet closely related areas of research, and suggest how those issues can be addressed by the course of further research (see Section 5.3). It has been regarded neither desired nor sensible to elaborate on the contents of all of the reviewed articles, nonetheless, the 20 examples shown above represent the character of the whole sample quite adequately, i.e., except for slight correlations, the trend of randomness continues all the way to the least-cited entry, number 155 presented (see Appendix A).

Ecosystem is an extremely ambiguous and multifaceted concept with myriad meanings that lead to an extensive amount of understandings and probably the most important conclusion that needs to be drawn up with regard to the previous literature is that the stigma that emerged in the BE is being passed on to the spun-off research domains. For that reason, it is important to realize that, if a scientist deliberately talks about BEs, she or he should do so by continuing within the realms of the narrative being told since 1993. The understanding of BEs should be an object of constructive critique and collectively shaped by mutual effort. Even when a researcher writes about Bes that are driven by IoT or other digital technologies, she or he still talks about BEs and should be familiar with the up-to-date status quo of the terminology (see Section 5.1). The author proposes that the most suitable conceptual point of departure for the interdisciplinary could be, for pragmatic reasons, the research lens adopted by Bogers et al. [9]. In this article, the authors re-link the dispersed merit and push the knowledge boundaries in a conceptually stable manner, while bridging the expanding gap between empirical findings and academia. On the other hand, when referring to an IoT-driven DBE as to a generally used expression, one should be clear and explicit about that and do not superficially mix it with established concepts.

Another important point to be highlighted is that the BE field (including DBEs and IoT-driven DBEs) has been increasingly expanding, but without the much-needed rigor or consistency. Moreover, when considering the current situation caused by COVID-19, the IoT infrastructure as well as the engagement in business ecosystems has become more important than ever [71,72]. Hence, it is legitimate to expect the exponential trend of growth to continue or even rise. The phenomenon that happens to occur as a result resembles the process universally known as inflation. Inflation can be defined as “an increase in prices over time, causing a reduction in the value of money”, according to the Cambridge Business English Dictionary. What can be observed in the area of DBE research driven by the IoT is fairly similar. The number of references is increasing; nonetheless, due to the poor conceptual rigor embedded, the value of the IoT-driven DBE research as a whole is on that premise is negatively impacted and eventually reduced. As discovered, a majority of the articles in this area are written by scholars and experts grounded in the branches of technology and engineering. Therefore, to mind the multifaceted nature of BEs, the researchers conducting interdisciplinary research should increase their efforts in pulling these fragments back together, so solid and consistent foundations for the increasingly important IoT-driven DBE research stream can be set. Having a heavily cited, but also heavily dispersed, concept may cause collapse of the field from the inside.

Naturally, this paper suffers from a number of limitations, with the key one being the format in which it is written. From the author’s point of view, an article that is based on a position paper only can ignite discussion and express an evidence-based point of view. From a broader perspective, this particular paper has one main mission—to highlight the importance of reinforcing theoretical rigor in cases when seemingly distant domain-specific foci collide in interdisciplinary arenas. It should not be understood as a critique of players but rather as a constructive feedback reflecting the state of the game. In general, achieving a balanced nexus of engineers and social scientist resembles a Sisyphean struggle. And exactly for that reason, it is necessary to communicate the significance of doing that in outlets targeting the groups that would not necessarily recognize the act of addressing this need as purposeful. Furthermore, it could be beneficial to explore the introduced ideas that are related to randomness in selection of concepts. Can a similar pattern be observed in any other interdisciplinary fields? Answering this straightforward question may help others to identify conceptual drawbacks in various interdisciplinary streams. Additionally, again, it is important to highlight that the embedded literature review is of a limited scope. Its purpose has been to prove the existence of the legacy scheme inherited by the IoT-driven DBE research from its BE antecedents. Nonetheless, as it does not go into detail, its authority should also be considered tentative, creating room for conducting a well-rounded and well-bounded review of literature that expands in volume every day. Finally, the scholars are encouraged to engage in academic debates concerning interdisciplinarity. Usually, social scientists address the issue of interdisciplinarity. This is for one simple reason—Engineers just want to solve the problem. Therefore, it might be salutary to build the bridges from both sides of the trench so the engineering scholars can also find them useful.

## Figures and Tables

**Table 1:** Overview of the key BE definitions related to Section 2.

Article	Definition
The Death of Competition: Leadership and Strategy in the Age of Business Ecosystems by J.F. Moore (1996)	“An economic community supported by a foundation of interacting organizations and individuals—The organisms of the business world. This economic community produces goods and services of value to customer, who themselves are members of the ecosystem. The member organisms also include suppliers, lead producers, competitors and other stakeholders. Over time, they coevolve their capabilities and roles, and tend to align themselves with the directions set by one or more central companies. Those companies holding leadership roles may change over time, but the function of ecosystem leader is valued by the community because it enables members to move toward shared visions to align their investments and to find mutually supportive roles”
Ecosystem as Structure: An Actionable Construct for Strategy by R. Adner (2016)	“The ecosystem is defined by the alignment structure of the multilateral set of partners that need to interact in order for a focal value proposition to materialize”.
Towards a theory of ecosystems by M.G. Jacobides, C. Cennamo and A. Gawer (2018)	“An ecosystem is a set of actors with varying degrees of multilateral, nongeneric complementarities that are not fully hierarchically controlled”.
What Is an Ecosystem? Incorporating 25 Years of Ecosystem Research by M. Bogers, J. Sims and J. West (2019)	“An interdependent network of self-interested actors jointly creating value”

**Table 2:** Systematic literature review process.

Phases	Stages
Planning the review	Identification of the need for a review
	Development of a review protocol.
Conducting the review	Identification of research
	Selection of primary studies
	Study quality assessment
	Data extraction & monitoring
	Data synthesis
Reporting the review	(Self-containing)

**Table 3:** Overview of literature review searches.

	Primary Queries	Total Entries	B&M Literature	Time Span
1st search	(“ecosystem”) OR (“business ecosystem”) OR (“digital business ecosystem”)	408,021	7092	1993–16 September, 2020
2nd search	(“internet of things”) OR (“IoT”)	88,187	3883	1999–16 September, 2020
3rd search	(“ecosystem” OR “business ecosystem” OR “digital business ecosystem”) AND (“internet of things” OR “IoT”)	2232	155	1999–16 September, 2020

## Appendix A. Total List of All 155 Relevant Peer-Reviewed IoT/BE & DBE Publications in the Field of Business and Management Adapted from the Scopus Database (3rd Search Round)

1. Gretzel, U.; Sigala, M.; Xiang, Z.; Koo, C. Smart tourism: Foundations and developments.* Electron. Mark.* **2015**, *25*, 179–188, doi:10.1007/s12525-015-0196-8.
2. Santoro, G.; Vrontis, D.; Thrassou, A.; Dezi, L. The Internet of Things: Building a knowledge management system for open innovation and knowledge management capacity.* Technol. Forecast. Soc. Chang.* **2018**, *136*, 347–354, doi:10.1016/j.techfore.2017.02.034.
3. Scuotto, V.; Ferraris, A.; Bresciani, S. Internet of Things: Applications and challenges in smart cities: A case study of IBM smart city projects.* Bus. Process Manag. J.* **2016**, *22*, 357–367, doi:10.1108/BPMJ-05-2015-0074.
4. Del Chiappa, G.; Baggio, R. Knowledge transfer in smart tourism destinations: Analyzing the effects of a network structure.* J. Destin. Mark. Manag.* **2015**, *4*, 145–150, doi:10.1016/j.jdmm.2015.02.001.
5. Rong, K.; Hu, G.; Lin, Y.; Shi, Y.; Guo, L. Understanding business ecosystem using a 6C framework in Internet-of-Things-based sectors.* Int. J. Prod. Econ.* **2015**, *159*, 41–55, doi:10.1016/j.ijpe.2014.09.003.
6. Buhalis, D.; Harwood, T.; Bogicevic, V.; Viglia, G.; Beldona, S.; Hofacker, C. Technological disruptions in services: Lessons from tourism and hospitalit.* J. Serv. Manag.* **2019**, *30*, 484–506, doi:10.1108/JOSM-12-2018-0398.
7. Balandina, E.; Balandin, S.; Koucheryavy, Y.; Mouromtsev, D. IoT Use Cases in Healthcare and Tourism. In Proceedings of the17th IEEE Conference on Business Informatics, Lisbon, Portugal, 13–16 July 2015; Volume 2, pp. 37–44, doi:10.1109/CBI.2015.16.
8. Papert, M.; Pflaum, A. Development of an Ecosystem Model for the Realization of Internet of Things (IoT) Services in Supply Chain Management.* Electron. Mark.* **2017**, *27*, 175–189, doi:10.1007/s12525-017-0251-8.
9. Shin, D.-H.; Jin Park, Y. Understanding the Internet of Things ecosystem: Multi-level analysis of users, society, and ecology.* Digit. Policy Regul. Gov.* **2017**, *19*, 77–100, doi:10.1108/DPRG-07-2016-0035.
10. Hein, A.; Weking, J.; Schreieck, M.; Wiesche, M.; Böhm, M.; Krcmar, H. Value co-creation practices in business-to-business platform ecosystems.* Electron. Mark.* **2019**, *29*, 503–518, doi:10.1007/s12525-019-00337-y.
11. Uchihira, N.; Ishimatsu, H.; Inoue, K. IoT service business ecosystem design in a global, competitive, and collaborative environment. In Proceedings of the PICMET 2016-Portland International Conference on Management of Engineering and Technology: Technology Management For Social Innovation, Honolulu, HI, USA, 4–8 September 2016; pp. 1195–1201, doi:10.1109/PICMET.2016.7806694.
12. Rajput, S.; Singh, S.P. Identifying Industry 4.0 IoT enablers by integrated PCA-ISM-DEMATEL approach.* Manag. Decis.* **2019**, *57*, 1784–1817, doi:10.1108/MD-04-2018-0378.
13. Madaan, N.; Ahad, M.A.; Sastry, S.M. Data integration in IoT ecosystem: Information linkage as a privacy threat.* Comput. Law Secur. Rev.* **2018**, *34*, 125–133, doi:10.1016/j.clsr.2017.06.007.
14. Harwood, T.; Garry, T. Internet of Things: Understanding trust in techno-service systems.* J. Serv. Manag.* **2017**, *28*, 442–475, doi:10.1108/JOSM-11-2016-0299.
15. Kummitha, R.K.R.; Crutzen, N. Smart cities and the citizen-driven internet of things: A qualitative inquiry into an emerging smart city.* Technol. Forecast. Soc. Chang.* **2019**, *140*, 44–53, doi:10.1016/j.techfore.2018.12.001.
16. Suri, K.; Gaaloul, W.; Cuccuru, A.; Gerard, S. Semantic framework for internet of things-aware business process development. In Proceedings of the 2017 IEEE 26th International Conference on Enabling Technologies: Infrastructure for Collaborative Enterprises (WETICE), Poznan, Poland, 21–23 June 2017; pp. 214–219, doi:10.1109/WETICE.2017.54.
17. Chandler, J.D.; Danatzis, I.; Wernicke, C.; Akaka, M.A.; Reynolds, D. How Does Innovation Emerge in a Service Ecosystem? *J. Serv. Res.* **2019**, *22*, 75–89, doi:10.1177/1094670518797479.
18. Tesch, J.F.; Brillinger, A.-S.; Bilgeri, D. Internet of Things Business Model Innovation And The Stage-Gate Process: An Exploratory Analysis.* Int. J. Innov. Manag.* **2017**, *2*, 1740002, doi:10.1142/S1363919617400023.
19. Leminen, S.; Rajahonka, M.; Westerlund, M.; Wendelin, R. The future of the Internet of Things: Toward heterarchical ecosystems and service business models.* J. Bus. Ind. Mark.* **2018**, *33*, 749–767, doi:10.1108/JBIM-10-2015-0206.
20. Settembre, M. Towards a hyper-connected world. In Proceedings of the 2012 15th International Telecommunications Network Strategy and Planning Symposium (NETWORKS), Rome, Italy, 15–18 October 2012; doi:10.1109/NETWKS.2012.6381667.
21. Manzoor, A.; Liyanage, M.; Braeke, A.; Kanhere, S.S.; Ylianttila, M. Blockchain based proxy re-encryption scheme for secure IoT data sharing. In Proceedings of the 2019-IEEE International Conference on Blockchain and Cryptocurrency (ICBC), Seoul, Korea, 14–17 May 2019; pp. 99–103, doi:10.1109/BLOC.2019.8751336.
22. Kolloch, M.; Dellermann, D. Digital innovation in the energy industry: The impact of controversies on the evolution of innovation ecosystems.* Technol. Forecast. Soc. Chang.* **2018**, *136*, 254–264, doi:10.1016/j.techfore.2017.03.033.
23. Brad, S.; Murar, M.; Brad, E. Design of smart connected manufacturing resources to enable changeability, reconfigurability and total-cost-of-ownership models in the factory-of-the-future.* Int. J. Prod. Res.* **2018**, *56*, 2269–2291, doi:10.1080/00207543.2017.1400705.
24. Tolstykh, T.; Shkarupeta, E.; Kostuhin, Y.; Zhaglovskaya, A. Key factors of manufacturing enterprises development in the context of industry 4.0. In Proceedings of the 31st International Business Information Management Association Conference (IBIMA): Innovation Management and Education Excellence through Vision, Milan, Italy, 25–26 April 2018; pp. 4747–4757.
25. Bullinger, H.-J.; Neuhüttler, J.; Nägele, R.; Woyke, I. Collaborative development of business models in Smart Service Ecosystems. In Proceedings of the 2017-Portland International Conference on Management of Engineering and Technology (PICMET): Technology Management for the Interconnected World, Portland, OR, USA, 9–13 July 2017; pp. 1–9, doi:10.23919/PICMET.2017.8125479.
26. Chouk, I.; Mani, Z. Factors for and against resistance to smart services: Role of consumer lifestyle and ecosystem related variables.* J. Serv. Mark.* **2019**, *33*, 449–462, doi:10.1108/JSM-01-2018-0046.
27. Leminen, S.; Rajahonka, M.; Westerlund, M. Actors in the emerging internet of things ecosystems.* Int. J. E-Serv. Mob. Appl.* **2017**, *9*, 57–75, doi:10.4018/IJESMA.2017010104.
28. Ermolayev, V.; Akerkar, R.; Terziyan, V.; Cochez, M. Toward evolving knowledge ecosystems for big data understanding*. Big Data Comput.* **2013**, 3–55, doi:10.1201/b16014.
29. Wöhrer, M.; Zdun, U. Design Patterns for Smart Contracts in the Ethereum Ecosystem. In Proceedings of the 2018 IEEE International Conference on Internet of Things (iThings) and IEEE Green Computing and Communications (GreenCom) and IEEE Cyber, Physical and Social Computing (CPSCom) and IEEE Smart Data (SmartData), Halifax, NS, Canada, 30 July–3 August 2018; pp. 1513–1520, doi:10.1109/Cybermatics_2018.2018.00255.
30. Uchibeke, U.U.; Schneider, K.A.; Kassani, S.H.; Deters, R. Blockchain Access Control Ecosystem for Big Data Security. In Proceedings of the 2018 IEEE International Conference on Internet of Things (iThings) and IEEE Green Computing and Communications (GreenCom) and IEEE Cyber, Physical and Social Computing (CPSCom) and IEEE Smart Data (SmartData), Halifax, NS, Canada, 30 July–3 August 2018; pp. 1373–1378, doi:10.1109/Cybermatics_2018.2018.00236.
31. Weking, J.; Stöcker, M.; Kowalkiewicz, M.; Böhm, M.; Krcmar, H. Leveraging industry 4.0–A business model pattern framework*. Int. J. Prod. Econ.* **2020**, *225*, 107588, doi:10.1016/j.ijpe.2019.107588.
32. Benbya, H.; Nan, N.; Tanriverdi, H.; Yoo, Y. Complexity and information systems research in the emerging digital world*. MIS Q. Manag. Inf. Syst.* **2020**, *44*, 1–17, doi:10.25300/MISQ/2020/13304.
33. Leminen, S.; Rajahonka, M.; Wendelin, R.; Westerlund, M. Industrial internet of things business models in the machine-to-machine context*. Ind. Mark. Manag.* **2020**, *84*, 298–311, doi:10.1016/j.indmarman.2019.08.008.
34. Hakanen, E.; Rajala, R. Material intelligence as a driver for value creation in IoT-enabled business ecosystems*. J. Bus. Ind. Mark.* **2018**, *33*, 857–867, doi:10.1108/JBIM-11-2015-0217.
35. Hamidi, H.; Jahanshahifard, M. The role of the Internet of Things in the improvement and expansion of business*. J. Organ. End User Comput.* **2018**, *30*, 24–44, doi:10.4018/JOEUC.2018070102.
36. Montori, F.; Contigiani, R.; Bedogni, L. Is WiFi suitable for energy efficient IoT deployments? A performance study. In Proceedings of the 2017 IEEE 3rd International Forum on Research and Technologies for Society and Industry (RTSI), Modena, Italy, 11–13 September 2017; doi:10.1109/RTSI.2017.8065943.
37. Viana, T.; Zisman, A.; Bandara, A.K. Identifying Conflicting Requirements in Systems of Systems. In Proceedings of the 2017 IEEE 25th International Requirements Engineering Conference (RE), Lisbon, Portugal, 4–8 September 2017; pp. 436–441, doi:10.1109/RE.2017.48.
38. Agarwal, N.; Chauhan, S.; Kar, A.K.; Goyal, S. Role of human behaviour attributes in mobile crowd sensing: A systematic literature review*. Digit. Policy Regul. Gov.* **2017**, *19*, 56–73, doi:10.1108/DPRG-05-2016-0023.
39. Khedekar, D.C.; Truco, A.C.; Oteyza, D.A.; Huertas, G.F. Home Automation—A Fast-Expanding Market*. Thunderbird Int. Bus. Rev.* **2017**, *59*, 79–91, doi:10.1002/tie.21829.
40. Benitez, G.B.; Ayala, N.F.; Frank, A.G. Industry 4.0 innovation ecosystems: An evolutionary perspective on value cocreation*. Int. J. Prod. Econ.* **2020**, *228*, 107735, doi:10.1016/j.ijpe.2020.107735.
41. Rubaiee, S.; Cinar, S.; Yildirim, M.B. An Energy-Aware Multiobjective Optimization Framework to Minimize Total Tardiness and Energy Cost on a Single-Machine Nonpreemptive Scheduling*. Ieee Trans. Eng. Manag.* **2019**, *66*, 699–714, doi:10.1109/TEM.2018.2846627.
42. Zdravković, M.; Zdravković, J.; Aubry, A.; Moalla, N.; Guedria, W.; Sarraipa, J. Domain framework for implementation of open IoT ecosystems*. Int. J. Prod. Res.* **2018**, *56*, 2552–2569, doi:10.1080/00207543.2017.1385870.
43. Ferreira, F.; Faria, J.; Azevedo, A.; Marques, A.L. Product lifecycle management enabled by industry 4.0 technology*. Adv. Transdiscipl. Eng.* **2016**, *3*, 349–354, doi:10.3233/978-1-61499-668-2-349.
44. Toivanen, T.; Mazhelis, O.; Luoma, E. Network analysis of platform ecosystems: The case of internet of things ecosystem*. Lect. Notes Bus. Inf. Process.* **2015**, *210*, 30–44, doi:10.1007/978-3-319-19593-3_3.
45. Abadi, F.A.; Ellul, J.; Azzopardi, G. The Blockchain of Things, beyond Bitcoin: A Systematic Review. In Proceedings of the 2018 IEEE International Conference on Internet of Things (iThings) and IEEE Green Computing and Communications (GreenCom) and IEEE Cyber, Physical and Social Computing (CPSCom) and IEEE Smart Data (SmartData), Halifax, NS, Canada, 30 July–3 August 2018; pp. 1666–1672, doi:10.1109/Cybermatics_2018.2018.00278.
46. Benomar, Z.; Bruneo, D.; Distefano, S.; Elbaamrani, K.; Idboufker, N.; Longo, F.; Merlino, G.; Puliafito, A.Extending openstack for cloud-based networking at the edge. In Proceedings of the 2018 IEEE International Conference on Internet of Things (iThings) and IEEE Green Computing and Communications (GreenCom) and IEEE Cyber, Physical and Social Computing (CPSCom) and IEEE Smart Data (SmartData), Halifax, NS, Canada, 30 July–3 August 2018; pp. 162–169, doi:10.1109/Cybermatics_2018.2018.00058.
47. Marchiori, M. Bins with Eyes: Towards a More Efficient Urban Ecosystem. In Proceedings of the 2018 IEEE International Conference on Internet of Things (iThings) and IEEE Green Computing and Communications (GreenCom) and IEEE Cyber, Physical and Social Computing (CPSCom) and IEEE Smart Data (SmartData), Halifax, NS, Canada, 30 July–3 August 2018; pp. 419–426, doi:10.1109/Cybermatics_2018.2018.00096.
48. Vătămănescu, E.-M.; Alexandru, V.-A. Beyond Innovation: The Crazy New World of Industrial Mash-ups*. Knowl. Manag. Organ. Learn.* **2018**, *6*, 271–285, doi:10.1007/978-3-319-66890-1_14.
49. Hein, A.; Böhm, M.; Krcmar, H. Platform configurations within information systems research: A literature review on the example of IoT platforms. In Proceedings of MKWI 2018-Multikonferenz Wirtschaftsinformatik 2018, Lüneburg, Germany, 6–9 March 2018; pp. 465–476.
50. Chochliouros, I.P.; Kostopoulos, A.; Spiliopoulou, A.S.; Dardamanis, A.; Neokosmidis, I.; Rokkas, T.; Goratti, L. Business and market perspectives in 5G networks. In Proceedings of 2017 Internet of Things Business Models, Users, and Networks, Copenhagen, Denmark, 23–24 November 2017; pp. 1–6, doi:10.1109/CTTE.2017.8260997.
51. Pochyla, M. Internet of things: Big challenge for enterprises. In Proceedings of the 11th International Conference on Strategic Management and its Support by Information Systems—SMSIS2015, Uherske Hradiste, Czechia, 21–22 May 2015; pp. 470–477.
52. Xu, L.D. The contribution of systems science to Industry 4.0*. Syst. Res. Behav. Sci.* **2020**, *37*, 618–631, doi:10.1002/sres.2705.
53. Seyedghorban, Z.; Tahernejad, H.; Meriton, R.; Graham, G. Supply chain digitalization: Past, present and future*. Prod. Plan. Control* **2020**, *31*, 96–114, doi:10.1080/09537287.2019.1631461.
54. Borgogno, O.; Colangelo, G. Data sharing and interoperability: Fostering innovation and competition through APIs*. Comput. Law Secur. Rev.* **2019**, *35*, 105314, doi:10.1016/j.clsr.2019.03.008.
55. Petrik, D.; Herzwurm, G. IIoT ecosystem development through boundary resources: A siemens mind sphere case study. In Proceedings of the 2nd ACM SIGSOFT International Workshop on Software-Intensive Business: Start-ups, Platforms, and Ecosystems, Tallin, Estonia, 26 August 2019; pp. 1–6, doi:10.1145/3340481.3342730.
56. Casadei, R.; Tsigkanos, C.; Viroli, M.; Dustdar, S. Engineering resilient collaborative edge-enabled IoT. In Proceedings of the 2019 IEEE International Conference on Services Computing (SCC), Milan, Italy, 8–13 July 2019; pp. 36–45, doi:10.1109/SCC.2019.00019.
57. Zanzi, L.; Cirillo, F.; Sciancalepore, V.; Giust, F.; Costa-Perez, X.; Mangiante, S.; Klas, G. Evolving multi-access edge computing to support enhanced IoT deployments*. IEEE Commun. Stand. Mag.* **2019**, *3*, 26–34, doi:10.1109/MCOMSTD.2019.1800009.
58. Puranik, V.; Sharmila, Ranjan, A.; Kumari, A. Automation in Agriculture and IoT. In Proceedings of the 2019 4th International Conference on Internet of Things: Smart Innovation and Usages (IoT-SIU), Ghaziabad, India, 18–19 April 2019; doi:10.1109/IoT-SIU.2019.8777619.
59. Armstrong, M.P.; Wang, S.; Zhang, Z. The Internet of Things and fast data streams: Prospects for geospatial data science in emerging information ecosystems*. Cartogr. Geogr. Inf. Sci.* **2019**, *46*, 39–56, doi:10.1080/15230406.2018.1503973.
60. Savastano, M.; Amendola, C.; D’Ascenzo, F. How digital transformation is reshaping the manufacturing industry value chain: The new digital manufacturing ecosystem applied to a case study from the food industry*. Lect. Notes Inf. Syst. Organ.* **2018**, *24*, 127–142, doi:10.1007/978-3-319-62636-9_9.
61. Oppitz, M.; Tomsu, P. Inventing the Cloud Century: How Cloudiness Keeps Changing Our Life, Economy and Technology; Springer: Berlin/Heidelberg, Germany, 2017; pp. 1–609, doi:10.1007/978-3-319-61161-7.
62. Markendahl, J.; Lundberg, S.; Kordas, O.; Movin, S. On the role and potential of IoT in different industries: Analysis of actor cooperation and challenges for introduction of new technology. In Proceedings of the 2017 Internet of Things Business Models, Users, and Networks, Copenhagen, Denmark, 23–24 November 2017; pp. 1–8, doi:10.1109/CTTE.2017.8260988.
63. Schreieck, M.; Hakes, C.; Wiesche, M.; Krcmar, H. Governing platforms in the internet of things*. Lect. Notes Bus. Inf. Process.* **2017**, *304*, 32–46, doi:10.1007/978-3-319-69191-6_3.
64. Zimmermann, A.; Schmidt, R.; Sandkuhl, K.; Jugel, D.; Bogner, J.; Möhring, M. Multi-perspective digitization architecture for the internet of things*. Lect. Notes Bus. Inf. Process.* **2017**, *263*, 289–298, doi:10.1007/978-3-319-52464-1_26.
65. Makinen, S.J. Internet-of-things disrupting business ecosystems: A case in home automation. In Proceedings of the 2014 IEEE International Conference on Industrial Engineering and Engineering Management, Bandar Sunway, Malaysia, 9–12 December 2014; pp. 1467–1470, doi:10.1109/IEEM.2014.7058882.
66. Zhu, L.; Cai, H.; Jiang, L. Minson: A business process self-adaptive framework for smart office based on multi-agent. In Proceedings of the 11th IEEE International Conference on E-Business Engineering, Guangzhou, China, 5–7 November 2014; pp. 31–37, doi:10.1109/ICEBE.2014.18.
67. Rasouli, M.R. An architecture for IoT-enabled intelligent process-aware cloud production platform: A case study in a networked cloud clinical laboratory*. Int. J. Prod. Res.* **2020**, *58*, 3765–3780, doi:10.1080/00207543.2019.1634847.
68. Zehir, S.; Zehir, M. Internet of Things in Blockchain Ecosystem from Organizational and Business Management Perspectives*. Contrib. Manag. Sci.* **2020**, 47–62, doi:10.1007/978-3-030-29739-8_3.
69. Song, R.; Vanthienen, J.; Cui, W.; Wang, Y.; Huang, L. Context-aware BPM using IoT-integrated context ontologies and IoT-enhanced decision models. In Proceedings of the 2019 IEEE 21st Conference on Business Informatics (CBI), Moscow, Russia, 15–17 July 2019; pp. 541–550, doi:10.1109/CBI.2019.00069.
70. Radermacher, W.J. *Official statistics 4.0: Verified Facts for People in the 21st Century*; Springer: Cham, Switzerland, 2019; pp. 1–158, doi:10.1007/978-3-030-31492-7.
71. Weber, P.; Morar, D.; Lasi, H. Transforming value chains into internet-based ecosystems: A testbed approach. In Proceedings of the PICMET 2018-Portland International Conference on Management of Engineering and Technology: Managing Technological Entrepreneurship: The Engine for Economic Growth, Honolulu, HI, USA, 19–23 August 2018, doi:10.23919/PICMET.2018.8481829.
72. Bobin, M.; Amroun, H.; Boukalle, M.; Anastassova, M.; Ammi, M. Smart Cup to Monitor Stroke Patients Activities during Everyday Life. In Proceedings of the 018 IEEE International Conference on Internet of Things (iThings) and IEEE Green Computing and Communications (GreenCom) and IEEE Cyber, Physical and Social Computing (CPSCom) and IEEE Smart Data (SmartData), Chengdu, China, 15–18 Deceber 2018; pp. 189–195, doi:10.1109/Cybermatics_2018.2018.00062.
73. Latif, Z.; Tunio, M.Z.; Pathan, Z.H.; Jianqiu, Z.; Ximei, L.; Sadozai, S.K. A review of policies concerning development of big data industry in Pakistan: Subtitle: Development of big data industry in Pakistan. In Proceedings of the 2018 international conference on computing, mathematics and engineering technologies (iCoMET), Sukkur, Pakistan, 3–4 March 2018; pp. 1–5, doi:10.1109/ICOMET.2018.8346315.
74. Tsaih, R.-H.; Hsu, C.C. Artificial intelligence in smart tourism: A conceptual framework. In Proceedings of the 18^th^ International Conference on Electronic Business (ICEB), Guilin, China, 2–6 December 2018; pp. 124–133.
75. Fabiano, N. The Internet of Things ecosystem: The blockchain and data protection issues*. ASTES J.* **2018**, *3*, 1–7, doi:10.25046/aj030201.
76. Iqbal, H.; Ma, J.; Mu, Q.; Ramaswamy, V.; Raymond, G.; Vivanco, D.; Zuena, J. Augmenting security of internet-of-things using programmable network-centric approaches: A position paper. In Proceedings of the 2017 26th International Conference on Computer Communications and Networks, Vancouver, BC, Canada, 31 July–3 August 2017; doi:10.1109/ICCCN.2017.8038504.
77. Karapantelakis, A.; Markendahl, J. Challenges for ICT business development in intelligent transport systems. In Proceedings of the 2017 Internet of Things Business Models, Users, and Networks, Copenhagen, Denmark, 23–24 November 2017; pp. 1–6, doi:10.1109/CTTE.2017.8260931.
78. Sandberg, J.; Holmström, J.; Lyytinen, K. Digitization and phase transitions in platform organizing logics: Evidence from the process automation industry*. MIS Q. Manag. Inf. Syst.* **2020**, *44*, 129–153, doi:10.25300/MISQ/2020/14520.
79. Tan, B.; Anderson, E.G.; Parker, G.G., Jr. Platform pricing and investment to drive third-party value creation in two-sided networks*. Inf. Syst. Res.* **2020**, *31*, 217–239, doi:10.1287/ISRE.2019.0882.
80. Lockl, J.; Schlatt, V.; Schweizer, A.; Urbach, N.; Harth, N. Toward Trust in Internet of Things Ecosystems: Design Principles for Blockchain-Based IoT Applications. *IEEE Trans. Eng. Manag.* **2020**, doi:10.1109/TEM.2020.2978014.
81. *Rocha 2020,* C.; Fernandes Narcizo, C.; Gianotti, E. Internet of management artifacts: Internet of things architecture for business model renewal*. Int. J. Innov. Technol. Manag.* **2019**, *16*, 1950062, doi:10.1142/S0219877019500627.
82. Aleksandrova, E.; Vinogradova, V.; Tokunova, G. Integration of digital technologies in the field of construction in the Russian Federation*. Eng. Manag. Prod. Serv.* **2019**, *11*, 38–47, doi:10.2478/emj-2019-0019.
83. Weber, P.; Hiller, S.; Lasi, H. Design and evaluation of an approach to generate cross-domain value scenarios in the context of the industrial internet of things: A capability-based approach. In Proceedings of the PICMET 2019-Portland International Conference on Management of Engineering and Technology: Technology Management in the World of Intelligent Systems, Portland, OR, USA, 25–29 August 2019; doi:10.23919/PICMET.2019.8893687.
84. Soni, D.; Sharma, V.; Srivastava, D. Optimization of security issues in adoption of cloud ecosystem. In Proceedings of the 2019 4th International Conference on Internet of Things: Smart Innovation and Usages, Ghaziabad, India, 18–19 April 2019; doi:10.1109/IoT-SIU.2019.8777670.
85. Poluri, M.; Kumar, A.; Allam, S.; Kiran, K.V.D. IoT ecosystem with blockchain and smart contracts*. Int. J. Recent Technol. Eng.* **2019**, *7*, 638–641.
86. Petrik, D.; Herzwurm, G. Towards an understanding of IIoT ecosystem evolution-mindsphere case study*. Lect. Notes Bus. Inf. Process.* **2019**, *370*, 46–54, doi:10.1007/978-3-030-33742-1_5.
87. Maydanova, S.; Ilin, I. Strategic approach to global company digital transformation. In Proceedings of the Proceedings of the 33rd International Business Information Management Association Conference, IBIMA 2019: Education Excellence and Innovation Management through Vision 2020, Granada, Spain, 10–11 April 2019; pp. 8818–8833.
88. Gary, R.F.; Marinakis, Y.; Majadillas, M.A.; White, R.; Walsh, S.T. Legitimate firms or hackers-who is winning the global cyber war? *Int. J. Technol. Intell. Plan.* **2019**, *12*, 297–314, doi:10.1504/IJTIP.2019.099243.
89. Lepoutre, J.; Perez, Y.; Petit, M. Energy transition and electromobility: A review. In *The European Dimension of Germany’s Energy Transition: Opportunities and Conflicts*; Springer: Cham, Switzerland 2019; pp. 509–525, doi:10.1007/978-3-030-03374-3_29.
90. Schulz, K.F.; Freund, D. A multichain architecture for distributed supply chain design in industry 4.0*. Lect. Notes Bus. Inf. Process.* **2019**, *339*, 277–288, doi:10.1007/978-3-030-04849-5_25.
91. Yerpude, S.; Singhal, T.K.; Rathod, H.R. Achieving service process excellence with connected customer: A winning approach*. Int. J. Inf. Syst. Supply Chain Manag.* **2019**, *12*, 66–80, doi:10.4018/IJISSCM.2019010104.
92. Bai, G.; Zhao, L.; Wang, Z.E. Advantech: Evolution of its IoT ecosystem strategy*. Emerald Emerg. Mark. Case Stud.* **2018**, *8*, 1–28, doi:10.1108/EEMCS-06-2018-0131.
93. Al-Sayed, R.; Yang, J. Towards Chinese smart manufacturing ecosystem in the context of the one belt one road initiative*. J. Sci. Technol. Policy Manag.* **2018**, *11*, 291–310, doi:10.1108/JSTPM-02-2018-0012.
94. Chao, F.-C. Sustainability for the Holistic Ecosystem: Regulation Guide Designing for the Prevalent Technology Development of China Emerging Communications: Big Data, IoT, 5G etc. In Proceedings of 2018 IEEE International Symposium on Innovation and Entrepreneurship (TEMS-ISIE), Beijing, China, 30 March-1 April 2018; doi:10.1109/TEMS-ISIE.2018.8478529.
95. Lokshina, I.; Durkin, B.; Lanting, C. The IoT-and big data-driven data analysis services: KM, implications and business opportunities*. Int. J. Knowl. Manag.* **2018**, *14*, 88–107, doi:10.4018/IJKM.2018100106.
96. Stanley, U.; Nkordeh, N.; Olu, V.M.; Bob-Manuel, I. IoT and 5G: The interconnection. In Proceedings of the 8th International Conference on Industrial Engineering and Operations Management, Bandung, Indonesia, 6–8 March 2018; pp. 374–386.
97. Hefnawy, A.; Bouras, A.; Cherifi, C. Relevance of lifecycle management to smart city development*. Int. J. Prod. Dev.* **2018**, *22*, 351–376, doi:10.1504/IJPD.2018.093430.
98. Degrande, T.; Vannieuwenborg, F.; Verbrugge, S.; Colle, D. Multi-sided Platforms for the Internet of Things*. Lect. Notes Bus. Inf. Process.* **2018**, *319*, 372–381, doi:10.1007/978-3-319-94214-8_28.
99. Fitzgerald, B.; Stol, K.-J. The future of software development methods. In *The Routledge Companion to Management Information Systems*; Routledge: Abingdon, UK; 2017; pp. 125–137, doi:10.4324/9781315619361.
100. Lopez-Martin, A.; Carvajal, R.G.; Cortés, P. Smart ecosystem for a sustainable, safe and integrated freight transport. In Proceedings of the 2016 IEEE International Conference on Emerging Technologies and Innovative Business Practices for the Transformation of Societies, Mauritius, Mauritius, 3–6 August 2016; pp. 143–146, doi:10.1109/EmergiTech.2016.7737327.
101. Olsson, H.H.; Bosch, J.; Katumba, B. User dimensions in ‘internet of things’ systems: The UDIT model*. Lect. Notes Bus. Inf. Process.* **2016**, *240*, 161–168, doi:10.1007/978-3-319-40515-5_13.
102. Yao, Y.; Yen, B.; Yip, A. Examining the effects of the internet of things (IoT) on e-commerce: Alibaba case study. In Proceedings of the 15th International Conference on Electronic Business (ICEB), Hong Kong, China 2015; pp. 247–257.
103. Subramaniam, M. Digital ecosystems and their implications for competitive strategy*. J. Organ. Des.* **2020**, *9*, 12, doi:10.1186/s41469-020-00073-0.
104. Pardo, C.; Ivens, B.S.; Pagani, M. Are products striking back? The rise of smart products in business markets*. Ind. Mark. Manag.* **2020**, *90*, 205–220, doi:10.1016/j.indmarman.2020.06.011.
105. Kahle, J.H.; Marcon, É.; Ghezzi, A.; Frank, A.G. Smart Products value creation in SMEs innovation ecosystems*. Technol. Forecast. Soc. Chang.* **2020**, *156*, 120024, doi:10.1016/j.techfore.2020.120024.
106. Demertzis, K.; Rantos, K.; Drosatos, G. A dynamic intelligent policies analysis mechanism for personal data processing in the iot ecosystem*. Big Data Cogn. Comput.* **2020**, *4*, 9, doi:10.3390/bdcc4020009.
107. Dhieb, N.; Ghazzai, H.; Besbes, H.; Massoud, Y. Scalable and Secure Architecture for Distributed IoT Systems. In Proceedings of the 2020 IEEE Technology and Engineering Management Conference, Detroit, MI, USA, 3–6 June 2020; doi:10.1109/TEMSCON47658.2020.9140108.
108. Muthuraman, S. Digital business models for sustainability*. Gedrag En Organ.* **2020**, *33*, 1095–1102, doi:10.37896/GOR33.02/115.
109. Vinod, V.M.; Mekala, V.; Abinaya, S.; Srinivas, A.; Arun, S. A customizable cartographic air pollution monitoring system*. Int. J. Sci. Technol. Res.* **2020**, *9*, 1675–1678.
110. Asthana, S.; Dwivedi, A. Performance measurement of India-based third party logistics sector: An empirical study of user versus provider perspectives*. Prod. Plan. Control* **2020**, *31*, 259–272, doi:10.1080/09537287.2019.1631467.
111. Jenweeranon, P. Thai regulatory approaches to technology-driven innovation in financial services*. Perspectives in Law, Business and Innovation*; Springer: Singapore, Singapore; 2020; pp. 97–114, doi:10.1007/978-981-15-5819-1_6.
112. Interview: Creating knowledge collaboratively to forge a richer society tomorrow-an innovation ecosystem to spearhead social transformation. In *Society 5.0: A People-centric Super-smart Society*; Springer: Singapore, 2020; pp. 145–154, doi:10.1007/978-981-15-2989-4_7.
113. Raff, S.; Wentzel, D.; Obwegeser, N. Smart Products: Conceptual Review, Synthesis, and Research Directions. *J. Prod. Innov. Manag.* **2020**, doi:10.1111/jpim.12544.
114. Jha, S.; Khanna, P. Study of enhancing employee engagement at workplace by adopting internet of things*. Int. J. Bus. Syst. Res.* **2020**, *14*, 341–361, doi:10.1504/IJBSR.2020.108282.
115. Poma, L.; Al Shawwa, H.; Maini, E. Industry 4.0 and big data: Role of government in the advancement of enterprises in Italy and UAE*. Int. J. Bus. Perform. Manag.* **2020**, *21*, 261–289, doi:10.1504/IJBPM.2020.108317.
116. Petrik, D.; Herzwurm, G. Complementor satisfaction with boundary resources in iiot ecosystems*. Lect. Notes Bus. Inf. Process.* **2020**, *389*, 351–366, doi:10.1007/978-3-030-53337-3_26.
117. Tyagi, A.K.; Abraham, A. Internet of things: Future challenging issues and possible research directions*. Int. J. Comput. Inf. Syst. Ind. Manag. Appl.* **2020**, *12*, 113–124.
118. Zimmermann, A.; Schmidt, R.; Jugel, D.; Möhring, M. Evolution of Enterprise Architecture for Intelligent Digital Systems*. Lect. Notes Bus. Inf. Process.* **2020**, *385*, 145–153, doi:10.1007/978-3-030-50316-1_9.
119. Lardo, A.; Mancini, D.; Paoloni, N.; Russo, G. The perspective of capability providers in creating a sustainable I4.0 environment. *Manag. Decis.* **2020**, doi:10.1108/MD-09-2019-1333.
120. Yang, X.; Cao, D.; Chen, J.; Xiao, Z.; Daowd, A. AI and IoT-based collaborative business ecosystem: A case in Chinese fish farming industry*. Int. J. Technol. Manag.* **2020**, *82*, 151–171, doi:10.1504/IJTM.2020.107856.
121. Wamanrao, A.; Raibagkar, R.L. A novel IoT architecture-based healthcare monitoring system*. Int. J. Emerg. Technol.* **2020**, *11*, 140–145.
122. Yano, M.; Dai, C.; Masuda, K.; Kishimoto, Y. Creation of blockchain and a new ecosystem. In *Blockchain and Crypt Currency;* Economics Law, and Institutions in Asia Pacific; Springer: Singapore, 2020; pp. 1–19, doi:10.1007/978-981-15-3376-1_1.
123. Ray, I.; Kar, D.M.; Peterson, J.; Goeringer, S. Device identity and trust in IoT-sphere forsaking cryptography. In Proceedings of the 5th IEEE International Conference on Collaboration and Internet Computing, Los Angeles, California, USA, 12–14 December 2019; pp. 204–213, doi:10.1109/CIC48465.2019.00034.
124. Zheng, P.; Wang, Z.; Chen, C.-H. Smart product-service systems: A novel transdisciplinary sociotechnical paradigm*. Adv. Transdiscipl. Eng.* **2019**, *10*, 234–241, doi:10.3233/ATDE190128.
125. Bharathi, S.V. Forewarned is forearmed: Assessment of IoT information security risks using analytic hierarchy process*. Benchmarking* **2019**, *26*, 2443–2467, doi:10.1108/BIJ-08-2018-0264.
126. Koenig, F.; Found, P.; Kumar, M. Improving maintenance quality in airport baggage handling operations*. Total Qual. Manag. Bus. Excell.* **2019**, *30* (Suppl. 1), S35–S52, doi:10.1080/14783363.2019.1665772.
127. Stanciu, A.; Petrescu, M.; Petrescu, A.G.; Bîlcan, F.R. Cyberaccounting for the leaders of the future. In *Improving Business Performance through Innovation in the Digital Economy*; IGI Global: Hershey, PA, USA; 2019; pp. 58–69, doi:10.4018/978-1-7998-1005-6.ch005.
128. Ugolini, M.; Smith, E. A Closer Look to the Future of Smart Cities. In Proceedings of the CTTE FITCE 2019: Smart Cities and Information and Communication Technology, Ghent, Belgium, 25–27 September 2019; doi:10.1109/CTTE-FITCE.2019.8894827.
129. Alkan, D.P. Re-shaping business strategy in the era of digitization. In *Handbook of Research on Strategic Fit and Design in Business Ecosystems*; IGI Global: Hershey, PA, USA; 2019; pp. 76–97, doi:10.4018/978-1-7998-1125-1.ch004.
130. Kubo, H.; Okoso, K. Business ecosystem strategy using new hydroponic culture method. In Proceedings of the PICMET 2019-Portland International Conference on Management of Engineering and Technology: Technology Management in the World of Intelligent Systems, Portland, Oregon, USA, 25–29 August 2019; doi:10.23919/PICMET.2019.8893714.
131. Kasai, E. LPWA alliance strategy: Example of sigfox alliance in Japan. In Proceedings of the PICMET 2019-Portland International Conference on Management of Engineering and Technology: Technology Management in the World of Intelligent Systems, Portland, OR, USA, 25–29 August 2019; doi:10.23919/PICMET.2019.8893921.
132. Maurya, A.K.; Ahmad, S. IoT based model for smart monitoring of network related infrastructure using integrated IoT platform (Boltiot)*. Int. J. Sci. Technol. Res.* **2019**, *8*, 1764–1769.
133. Agostino Ardagna, C.; Asal, R.; Damiani, E.; El Ioini, N.; Pahl, C. Trustworthy IoT: An evidence collection approach based on smart contracts. In Proceedings of the 2019 IEEE International Conference on Services Computing (SCC), Milan, Italy, 8–13 July 2019; pp. 46–50, doi:10.1109/SCC.2019.00020.
134. Sutar, S.H.; Jinila, Y.B. Congestion control for better performance of WSN based iot ecosystem using KHA mechanism*. Int. J. Recent Technol. Eng.* **2019**, *8*, 567–570, doi:10.35940/ijrte.B1104.0782S319.
135. Jell, T.; Baumgartner, C.; Broring, A.; Mitic, J. BIG IoT-Interconnecting IoT Platforms from different domains-Final Results. In Proceedings of the 2019 IEEE International Conference on Engineering, Technology and Innovation, Valbonne, France, 17–19 June 2019; doi:10.1109/ICE.2019.8792626.
136. Kuonen, D.; Loison, B. Production processes of official statistics and analytics processes augmented by trusted smart statistics: Friends or foes? *Stat. J. IAOS* **2019**, *35*, 615–622, doi:10.3233/SJI-190530.
137. Hari Kumar, N.; Baskaran, S. Machine learning: The Panacea for 5G complexities*. J. Ict Stand.* **2019**, *7*, 157–170, doi:10.13052/jicts2245-800X.726.
138. Baskaran, S.B.M. Internet of things security*. J. ICT Stand.* **2019**, *7*, 21–39, doi:10.13052/jicts2245-800X.712.
139. Rieger, C.; Kuchen, H. Towards Pluri-Platform Development: Evaluating a Graphical Model-Driven Approach to App Development Across Device Classes*. Lect. Notes Bus. Inf. Process.* **2019**, *347*, 36–66, doi:10.1007/978-3-030-28430-5_3.
140. Chen, Y.-J.; Lu, T.-J. Intellectual property strategy for the ecosystem of the internet of things.* Int. J. Technol. Manag.* **2019**, *80*, 212–240, doi:10.1504/IJTM.2019.100286.
141. Lomotey, R.K.; Sriramoju, S.; Orji, R. Machine-to-infrastructure middleware platform for data management in IoT.* Int. J. Bus. Process Integr. Manag.* **2019**, *9*, 90–106, doi:10.1504/IJBPIM.2019.099874.
142. Gupta, R.; Miyazaki, K.; Kajikawa, Y. Ingredients of successful emerging business ecosystems: Case of industrial IoT adoption. In Proceedings of the PICMET 2018-Portland International Conference on Management of Engineering and Technology: Managing Technological Entrepreneurship: The Engine for Economic Growth, Honolulu, HI, USA, 19–23 August 2018; doi:10.23919/PICMET.2018.8481854.
143. Nehru, Yudertha, A.; Hais, Y.R. A Design Framework for Real-time Heterogeneous Sensor Data Acquisition as a Basis of IoT Ecosystem Development. In Proceedings of the 2018 International Conference on Information Technology Systems and Innovation, Padang, Indonesia, 22–26 October 2018; pp. 205–208, doi:10.1109/ICITSI.2018.8696027.
144. Gaur, A.S.; Budakoti, J.; Lung, C.-H. Design and performance evaluation of containerized microservices on edge gateway in mobile IoT. In Proceedings of the IEEE 2018 International Congress on Cybermatics: 2018 IEEE Conferences on Internet of Things, Green Computing and Communications, Cyber, Physical and Social Computing, Smart Data, Blockchain, Computer and Information Technology, iThings/GreenCom/CPSCom/SmartData/Blockchain/CIT, Halifax, NS, Canada, 30 July–3 August 2018; pp. 138–145, doi:10.1109/Cybermatics_2018.2018.00055.
145. Johnson, B.; Laszka, A.; Grossklags, J.; Moore, T. Economic Analyses of Security Investments on Cryptocurrency Exchanges. In Proceedings of the IEEE 2018 International Congress on Cybermatics, Halifax, NS, Canada, 30 July–3 August 2018; pp. 1253–1262, doi:10.1109/Cybermatics_2018.2018.00220.
146. Hirata, S. An empirical study on value creation of multi-product small-volume production through industry-academia collaboration. In Proceedings of the IEEE International Conference on Industrial Engineering and Engineering Management, Bangkok, Thailand, 16–19 December 2018; pp. 1738–1742, doi:10.1109/IEEM.2017.8290189.
147. Voronova, I.; Shatrevich, V.; Freimane, G. The impact of digital transformation on development of Latvian insurance companies’ digitalization strategies and shift of perception values. In Proceedings of the 32nd International Business Information Management Association Conference (IBIMA), Seville, Spain, 15–16 November 2018; pp. 5058–5069.
148. Vadera, S. A study on the growth of millennial entrepreneurs in India. In Proceedings of the 13th European Conference on Innovation and Entrepreneurship (ECIE), Aveiro, Portugal, 20–21 September 2018; pp. 831–837.
149. Ferreira, J.C.; Monteiro, V.; Afonso, J.L.; Martins, A.L.; Afonso, J.A. Mobile device sensing system for urban goods distribution logistics. In Proceedings of the 2017 IEEE International Conference on Service Operations and Logistics, and Informatics, Bari, Italy, 18–20 September 2017; pp. 187–192, doi:10.1109/SOLI.2017.8120992.
150. Wu, Q.; Wang, X.; Wang, X.; Li, Y.; Liang, Y.; Wang, Y. Construction of wheat’s photosynthetic carbon model. In Proceedings of the 6th International Conference on Agro-Geoinformatics, Agro-Geoinformatics, Faifax, WV, USA, 7–10 August 2017; doi:10.1109/Agro-Geoinformatics.2017.8047067.
151. Valsamakis, Y.; Savidis, A. Personal applications in the internet of things through visual end-user programming. In *Digital Marketplaces Unleashed*; Springer: Heidelberg, Germany, 2017; pp. 809–821, doi:10.1007/978-3-662-49275-8_71.
152. Hossain, M.I.; Markendahl, J. IoT-communications as a service: Actor roles on indoor wireless coverage. In Proceedings of the Joint 13th CTTE and 10th CMI Conference on Internet of Things-Business Models, Users, and Networks, Copenhagen, Denmark, 23–24 November 2017; pp. 1–7, doi:10.1109/CTTE.2017.8260933.
153. Ziyae, B.; Zali, M.R. A DNA business model for the internet of things: A value-creating ecosystem in Iranian small and medium-sized enterprises. In *Iranian Entrepreneurship: Deciphering the Entrepreneurial Ecosystem in Iran and in the Iranian Diaspora*; Springer: Cham, Switzerland; 2017; pp. 331–341, doi:10.1007/978-3-319-50639-5_20.
154. Kale, V. Creating Smart Enterprises: Leveraging Cloud, Big Data, Web, Social Media, Mobile and IoT Technologies; CRC Press: Boca Raton, FL, USA, 2017; pp. 1–380, doi:10.1201/9781315152455.
155. Lin, A.; Madden, A. Understanding the role of IoT technologies in supply ecosystems. In Proceedings of the 15th International Conference on Electronic Business (ICEB 2015), Hong Kong, China, 6–10 December 2015; pp. 484–491.
